# A Syst-OMICS Approach to Ensuring Food Safety and Reducing the Economic Burden of Salmonellosis

**DOI:** 10.3389/fmicb.2017.00996

**Published:** 2017-06-02

**Authors:** Jean-Guillaume Emond-Rheault, Julie Jeukens, Luca Freschi, Irena Kukavica-Ibrulj, Brian Boyle, Marie-Josée Dupont, Anna Colavecchio, Virginie Barrere, Brigitte Cadieux, Gitanjali Arya, Sadjia Bekal, Chrystal Berry, Elton Burnett, Camille Cavestri, Travis K. Chapin, Alanna Crouse, France Daigle, Michelle D. Danyluk, Pascal Delaquis, Ken Dewar, Florence Doualla-Bell, Ismail Fliss, Karen Fong, Eric Fournier, Eelco Franz, Rafael Garduno, Alexander Gill, Samantha Gruenheid, Linda Harris, Carol B. Huang, Hongsheng Huang, Roger Johnson, Yann Joly, Maud Kerhoas, Nguyet Kong, Gisèle Lapointe, Line Larivière, Stéphanie Loignon, Danielle Malo, Sylvain Moineau, Walid Mottawea, Kakali Mukhopadhyay, Céline Nadon, John Nash, Ida Ngueng Feze, Dele Ogunremi, Ann Perets, Ana V. Pilar, Aleisha R. Reimer, James Robertson, John Rohde, Kenneth E. Sanderson, Lingqiao Song, Roger Stephan, Sandeep Tamber, Paul Thomassin, Denise Tremblay, Valentine Usongo, Caroline Vincent, Siyun Wang, Joel T. Weadge, Martin Wiedmann, Lucas Wijnands, Emily D. Wilson, Thomas Wittum, Catherine Yoshida, Khadija Youfsi, Lei Zhu, Bart C. Weimer, Lawrence Goodridge, Roger C. Levesque

**Affiliations:** ^1^Institute for Integrative and Systems Biology, Université Laval, Québec CityQC, Canada; ^2^McGill University, MontréalQC, Canada; ^3^National Microbiology Laboratory, Public Health Agency of Canada, OttawaON, Canada; ^4^Laboratoire de Santé Publique du Québec, Sainte-Anne-de-BellevueQC, Canada; ^5^Université Laval, Québec CityQC, Canada; ^6^Institute of Food and Agricultural Sciences, University of Florida, GainesvilleFL, United States; ^7^Département de Microbiologie, Infectiologie et Immunologie, Université de Montréal, MontréalQC, Canada; ^8^Agriculture and Agri-Food Canada, SummerlandBC, Canada; ^9^Génome Québec Innovation Center, MontréalQC, Canada; ^10^Food Safety Engineering, Faculty of Land and Food Systems, University of British Columbia, VancouverBC, Canada; ^11^National Institute for Public Health and the EnvironmentBilthoven, Netherlands; ^12^Canadian Food Inspection Agency, HalifaxNS, Canada; ^13^Bureau of Microbial Hazards, Health Canada, OttawaON, Canada; ^14^UC Davis Food Science and Technology, DavisCA, United States; ^15^UC Davis School of Veterinary Medicine, DavisCA, United States; ^16^Canadian Food Inspection Agency, OttawaON, Canada; ^17^Food Science, University of Guelph, GuelphON, Canada; ^18^Department of Microbiology and Immunology, Faculty of Pharmacy, Mansoura UniversityMansoura, Egypt; ^19^Department of Microbiology and Immunology, Dalhousie University, HalifaxNS, Canada; ^20^Department of Biological Sciences, University of Calgary, CalgaryAB, Canada; ^21^Institute for Food Safety and Hygiene, University of ZurichZurich, Switzerland; ^22^Biological and Chemical Sciences, Wilfrid Laurier University, WaterlooON, Canada; ^23^Department of Food Science, Cornell University, IthacaNY, United States; ^24^College of Veterinary Medicine, The Ohio State University, ColumbusOH, United States

**Keywords:** *Salmonella*, foodborne pathogen, next-generation sequencing, bacterial genomics, phylogeny, antibiotic resistance, database

## Abstract

The *Salmonella* Syst-OMICS consortium is sequencing 4,500 *Salmonella* genomes and building an analysis pipeline for the study of *Salmonella* genome evolution, antibiotic resistance and virulence genes. Metadata, including phenotypic as well as genomic data, for isolates of the collection are provided through the *Salmonella* Foodborne Syst-OMICS database (SalFoS), at https://salfos.ibis.ulaval.ca/. Here, we present our strategy and the analysis of the first 3,377 genomes. Our data will be used to draw potential links between strains found in fresh produce, humans, animals and the environment. The ultimate goals are to understand how *Salmonella* evolves over time, improve the accuracy of diagnostic methods, develop control methods in the field, and identify prognostic markers for evidence-based decisions in epidemiology and surveillance.

## Importance of Foodborne *Salmonella* as a Model in Large-Scale Bacterial Genomics

*Salmonella enterica* is a foodborne bacterial pathogen having at least 2,600 serotypes (Gal-Mor et al., 2014)^1^ that contaminates a diversity of foods and is a leading cause of foodborne illnesses and mortality globally. In fact, there are an estimated 93.3 million cases of gastroenteritis due to non-typhoidal *Salmonella* infections each year, resulting in approximately 155,000 deaths (Majowicz et al., 2010). In Canada, non-typhoidal salmonellosis accounts for more than 88,000 cases of foodborne illness each year, and has among the highest incidence rate of any bacterial foodborne pathogen (Thomas et al., 2015). *S. enterica* is responsible for more than 50% of fresh produce-borne outbreaks, the highest number of foodborne outbreaks of any inspected food commodity in North America (Kozak et al., 2013). Because of its remarkable genomic diversity, *Salmonella* is found in complex environmental and ecological niches and survives in harsh environments for long periods (Podolak et al., 2010; Fatica and Schneider, 2011). Several research groups have identified relationships between some of the 2,557 *S. enterica* serotypes and specific foods, which suggests, that some food commodities act as reservoirs for particular serotypes (Kim, 2010; Jackson et al., 2013; Nuesch-Inderbinen et al., 2015).

*Salmonella* outbreaks are monitored with support from the PulseNet surveillance system in 86 countries^2^ (Ribot and Hise, 2016; Scharff et al., 2016). PulseNet Canada^3^ is a national surveillance system used to quickly identify and respond to foodborne disease outbreaks, centralized at the National Microbiology Laboratory in Winnipeg, MB, and working in close collaboration with a network of federal and provincial public health laboratories and epidemiologists. Still, despite the availability of thousands of sequenced genomes, knowledge of genome evolution integrated with transmission and epidemiology is limited for produce-related outbreaks.

Studies of *S. enterica* population structure in humans, animals, food and the environment are central to understand the biodiversity, evolution, ecology and epidemiology of this pathogen. However, studies describing the genetic structure of *Salmonella* populations are commonly based on isolates drawn overwhelmingly from clinical collections (Hoffmann et al., 2014). This approach has resulted in a limited view of *Salmonella*’s evolutionary history (D’costa et al., 2006; Perry and Wright, 2014). In *Salmonella* as in many other bacterial pathogens, there is limited knowledge on how genome content, rearrangements and the complement of genes including those acquired by horizontal gene transfer (HGT) contribute to strain-specific phenotypes, including virulence (Casadevall, 2017). Various studies have sought to resolve the population structure of *Salmonella* using complementary subtyping methods including pulsed-field gel electrophoresis (PFGE), multiple loci VNTR analysis (MLVA), 7-gene housekeeping schemes, whole-genome multi-locus sequence typing (wgMLST) profiles, pan- and core genome studies, and CRISPR analysis to define molecular signatures, pathogen subtypes and the potential for pathogenicity (Shariat and Dudley, 2014; Rouli et al., 2015; Liu et al., 2016). Next-generation sequencing (NGS) coupled with whole-genome comparison is well-positioned to become the gold standard subtyping method, as it offers previously unmatched resolution for phylogenetic analysis and rapid subtyping during investigation of food contamination and outbreaks (Ashton et al., 2016; Bekal et al., 2016).

## The Syst-OMICS Strategy

The application of genomics to infectious pathogens via WGS is transforming the practice of *Salmonella* diagnostics, epidemiology and surveillance. Genomic data are increasingly used to understand infectious disease epidemiology (Didelot et al., 2017). With rapidly falling costs and turnaround time, microbial WGS and analysis is becoming a viable strategy to identify the geographic origin of bacterial pathogens (Weedmark et al., 2015; Hoffmann et al., 2016). The objective of the Canadian-based international Syst-OMICS consortium is to sequence a minimum of 4,500 genomes, include the data in the *Salmonella* Foodborne Syst-OMICS database (SalFoS) at https://SalFoS.ibis.ulaval.ca/, share this information plus available metadata with Canadian federal and provincial regulators and the food industry, and develop pipelines to study these genomes. Genomics data will support molecular epidemiology and source attribution of outbreaks and has the potential for future genotypic antimicrobial susceptibility testing, as well as the identification of novel therapeutic targets and prognostic markers. Moreover, the large-scale genomics and evolutionary biology tools developed may lead to new strategies for countering not only *Salmonella* infections, but other pathogens as well (Little et al., 2012).

The Syst-OMICS project is based upon a systems approach (flowchart and screening method available in Supplementary File 1). First, the genome diversity of 4,500 isolates will be assessed using high-quality WGS, assembly, annotation and phylogeny. This data will be used for *in silico* serotyping (Yoshida et al., 2016), as well as analysis of virulence (Chen et al., 2012), antibiotic resistance (Jia et al., 2016) and mobilome gene content (Lanza et al., 2014). Based on this genomic data, a funnel-type model will be applied such that 300 isolates will be selected for *in vitro* high-throughput screening (HTS) in cell lines to determine attachment, adhesion, invasion and replication of each isolate (protocol adapted to 96-well plates from Forest et al., 2007). From the results, isolates will be categorized as being of high, medium, or low virulence. A limited number of those isolates will then be selected for further screening *in vivo* using a mouse model (Roy et al., 2007) and *in vitro* using gastrointestinal fermenter models (Kheadr et al., 2010; Le Blay et al., 2012). These data will identify isolates to represent the different levels of virulence that will be used to develop novel diagnostic and control tools. We propose to enhance food safety and lower the economic burden of salmonellosis through a farm-to-table systematic approach to control *Salmonella*, with a focus on new control methods in agricultural production, more specific diagnostics and improved bacterial subtyping methods to support investigation of foodborne outbreaks, as no single intervention is likely to produce meaningful and lasting effects.

## The *Salmonella* Foodborne Syst-OMICS Database (SalFoS)

*Salmonella* Foodborne Syst-OMICS database is an online web application that relies on a Mysql 5 database. It was designed not only to store data for the *Salmonella* strain collection but also to provide access to each isolate’s phenotypic, genomic, virulence, serotype, mobilome and epidemiological data. Different levels of access may be granted, but data modification is strictly reserved to the curators. It includes isolate identification, host, provider, date of isolation, geographical origin, phenotypic data, DNA extraction details, NGS information and genome assembly statistics. SalFoS currently contains NGS data and unpublished draft genomes from produce, human, animal and environmental isolates. Upon publication, draft genomes of SalFoS will become available at NCBI and EnteroBase^4^.

The SalFoS collection currently contains 2,498 entries for *Salmonella*, as well as for *Citrobacter*, *Hafnia* and *Proteus*, three genera often identified as false-positives by a number of *Salmonella* detection schemes. It includes previously described collections such as the unique *Salmonella* Genetic Stock Centre strains, described at http://people.ucalgary.ca/tilde{}kesander/. This collection was assembled with the aim of representing maximal genomic diversity.

## Sequencing 4,500 *Salmonella* Genomes: Objectives and Strategy

Our working hypothesis is that a very high-quality, large-scale bacterial genome database available through a user-friendly pipeline will have a major impact for epidemiology, diagnosis, prevention and treatment. By generating a comprehensive genome sequence database truly representative of the foodborne *Salmonella* population, we will: (1) assemble a large and representative strain collection, with associated genome data, useful for antimicrobial testing, identification of resistance markers, data mining for new therapeutic targets and development of machine learning strategies; (2) develop platforms and pipelines to manage and analyze this information, which will allow identification of prognostic markers, fast epidemiological tracking and reduction of socio-economic costs. We seek to develop user-friendly tools that will enable epidemiologists, microbiologists, clinicians and others to interpret genomic data, thus leading to informed decisions in cases of food contamination and outbreaks. The contamination of fresh produce by *Salmonella* will be addressed through the development of natural solutions to control the presence of *Salmonella* on fruits and vegetables as they are growing in the fields. New tests will also be developed so that fresh produce can be quickly and efficiently tested for the presence of *Salmonella* before being sold to consumers. In the context of outbreak investigation, the genomic data will be used to assess high-quality SNPs and core/whole genome MLST for their usefulness in genetic discrimination in addition to other emerging methods such as CRISPR and prophage sequence typing. As for outbreak investigation software, the National Microbiology Laboratory-Public Health Agency of Canada group has implemented the Integrated Rapid Infectious Disease Analysis project (IRIDA)^5^ and developed the SNVPhyl phylogenomics pipeline that is in use by PulseNet Canada for microbial genomic epidemiology (Petkau et al., 2016). A complementary system called the Metagenomics Computation and Analytics Workbench (MCAW) is being implemented as a computing service for food safety (Edlund et al., 2016; Weimer et al., 2016).

Sequencing for this project is performed on an Illumina MiSeq instrument (at the Plateforme d’Analyses Génomiques of the IBIS, Université Laval, Quebec City, QC, Canada), at a rate of 120 genomes per week, using 300 bp paired-end libraries, and with a median coverage of 45×. In order to perform core genome phylogenetic analysis, the pan-genome, i.e., the complete repertoire of genes of a species, is determined using a recently developed software capable of handling high-quality NGS data from thousands of genomes: Saturn V version 1.0^6^ (Jeukens et al., 2017). Additional analyses focus on genes implicated in virulence using comparative genomics predictions of confirmed and predicted virulence factors (Yang et al., 2008), and resistome identification based on the comprehensive antibiotic resistance database (CARD) (Mcarthur et al., 2013; Jia et al., 2016). A set of new reference *Salmonella* genomes representing maximal genomic diversity among foodborne pathogens will then be selected for PacBio Sequel sequencing to become fully assembled and annotated as a single circular chromosome.

## The Ibis Bioinformatics Pipeline for Genome Assembly

When working with hundreds or thousands of genomes, analysis software for assembly, annotation, statistics for quality control and selection of additional reference genomes is required to extract relevant information in an automated and reliable fashion with minimal human intervention. Ideally, this software should be platform independent and able to analyze sequence data directly without being tied to proprietary data formats. This insures maximal flexibility and reduces lag time to a minimum. We are currently using an integrated pipeline for *de novo* assembly of microbial genomes based on the A5 pipeline (Tritt et al., 2012). It was parallelized on a Silicon Graphics UV 300 using up to 120 cores to accommodate raw data from 120 genomes and provide assembly statistics as well as reference genome alignment metrics in as little as 2 h. This automated approach currently results in a median of 35 scaffolds per genome (median N50 = 462 kb).

## Phylogeny of *Salmonella*

**FIGURE 1:**
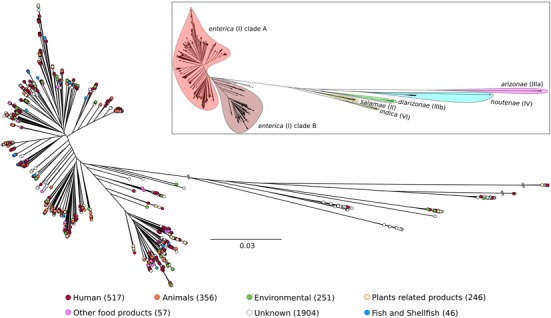
Unrooted maximum likelihood tree of 3,377 *Salmonella enterica* genomes based on 196,774 SNPs using FastTree 2.1.9 (1000 bootstraps). The six *S. enterica* subspecies names and specific epithets are indicated on the upper right tree. *S. enterica* subspecies *enterica* is split into two major lineages, clade A and clade B, as proposed by Timme et al. (2013). The two *S. bongori* (V) isolates contained in SalFoS were not included in the phylogenetic tree because they unnecessarily decrease resolution within the *S. enterica* subspecies. Number of genomes within each *S. enterica* subspecies are 3,235 *enterica* (2,648 in clade A and 587 in clade B), 51 *houtenae*, 32 *diarizonae*, 28 *salamae*, 17 *indica*, 8 *arizonae* and 6 with unknown subspecies. Tree tips were colored based on the source of each isolate. The number of isolates represented for each source category is shown between parentheses.

Once isolates from a given outbreak are sequenced, patterns of shared variations can be used to infer which isolates within the outbreak are most closely related to each other (e.g., Didelot et al., 2017). As a future strategy for the Syst-OMICS project, this could be applied to partially sampled and on-going *Salmonella* outbreaks. Here, as a first step in the study of *S. enterica* diversity and epidemiology, we used 3,377 genomes; 1,627 were from a collaboration with UC Davis (Bart C. Weimer), and 1,750 were part of SalFoS. All genomes with >100 scaffolds were eliminated; this filter typically removes the vast majority of low coverage (i.e., low quality) assemblies and mixed cultures. As our assembly pipeline also includes alignment on a suite of reference genomes, it is also possible to ensure that genomes used belong to *S. enterica*. The core (conserved) genome was identified with Saturn V, and consisted of 839 genes, which were used for phylogenetic analysis. This number of core genes, which seems small compared to other studies (2,882 core genes for 73 genomes from 2 subspecies, Leekitcharoenphon et al., 2012), is due to both the extensive diversity and the high number of genomes used. As depicted in **Figure 1**, this population of *S. enterica* strains could be divided into seven major groups. They correspond to *S. enterica* subspecies *enterica* clades A and B and a collection of branching subspecies previously defined as *salamae, arizonae, diarizonae, houtenae* and *indica.* The significant number of strains (3,377) included in our analysis and their wide-ranging sources (including environmental, human, animal and food) is essential to understand the diversity of *Salmonella* as a foodborne pathogen and in defining levels of virulence. The remarkable genomic diversity exhibited in **Figure 1** is thought to enable the colonization of a wide range of ecological niches. The *Salmonella* Syst-OMICS consortium will provide fine-scale analysis of this diversity via virulence factors, antibiotic resistance genes as well as complete core and accessory genomes.

## Linking SalFoS with the Comprehensive Antibiotic Resistance Database

The SalFoS database is intended to become an established platform for searching and comparing multiple genome sequences for *Salmonella* isolates. The database will also incorporate genome annotation and serotype prediction based on SISTR (Yoshida et al., 2016). Close attention to the links between specific genomic islands and patterns of SNPs in the core genome will help identify diagnostic sequences and SNP combinations for the development of new *Salmonella* subtyping methods with the highest resolution to date. This will be done using *de novo* island prediction with IslandViewer (Langille and Brinkman, 2009; Dhillon et al., 2015) as well as with gene presence-absence from SaturnV.

**FIGURE 2:**
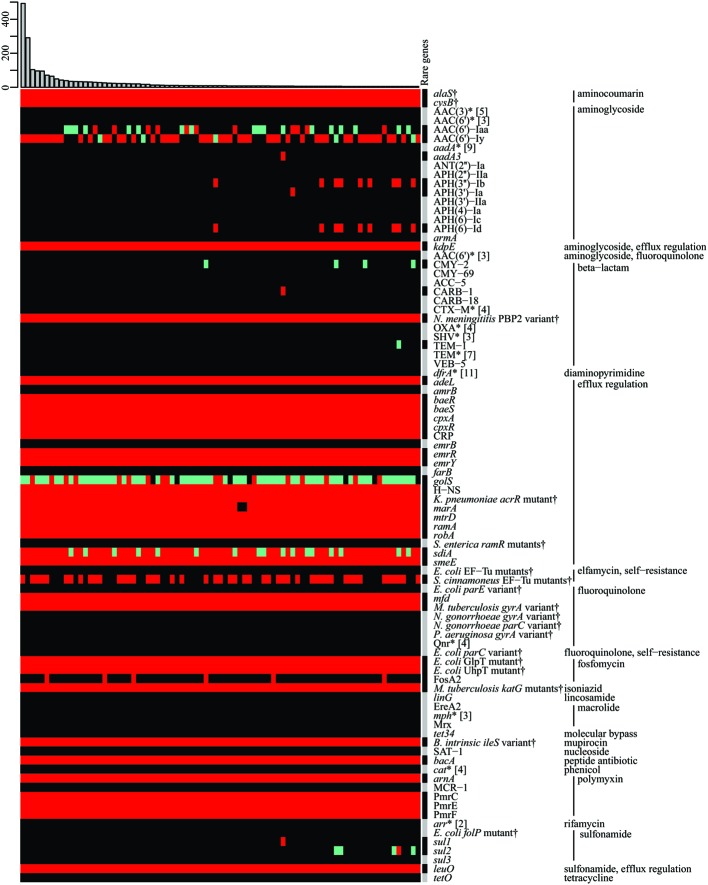
The resistome of 3,377 *Salmonella* strains. Gene, protein or variant presence were determined using the RGI-CARD (Mcarthur et al., 2013). Of 1,003 unique resistomes, only those present in at least five strains are shown; the histogram at the top represents absolute frequency. Other resistomes are condensed in the “Rare genes” column. AMR genes and variants are grouped by antibiotic family or function. Genes encoding efflux pumps, which are generally conserved, have been removed for figure clarity. Green: perfect match to a gene in the CARD, red: similar to a gene in the CARD, according to curated homology cut-offs, gray: perfect match and/or similar to a gene in the CARD, black: no match in the CARD, ^∗^(wildcard) represents multiple forms (exact number between square brackets) of the same gene or protein, ^†^specific variants conferring resistance (protein variant models).

As an additional feature, we routinely determine the resistome of the genomes in SalFoS, i.e., the genes and variants likely involved in antibiotic resistance. This is done using the Resistance Gene Identifier (RGI) available with the CARD (Mcarthur et al., 2013; Jia et al., 2016), at http://arpcard.mcmaster.ca/. **Figure 2** summarizes the resistomes of 3,377 genomes. In fact, the original dataset contained 1,003 unique resistomes, composed of various combinations of 195 different genes and variants. Despite this impressive diversity, the most striking feature shown in **Figure 2** is that the two most frequently observed resistomes, which are extremely similar, account for 23% of the strains. They are therefore highly conserved and warrant further investigation. These results will be exploited to study and understand the pool of resistance genes present in *Salmonella* strains, with a focus on strains found in fresh produce, to understand the links between foodborne *Salmonella* and environmental strains with respect to resistance genes.

## Linking Genomic and Clinical Data

It will be essential to match phenotypic, epidemiological and available clinical *Salmonella* data (antibiotic resistance, virulence, and anonymized clinical observations) to the genomic data produced. We will categorize metadata in SalFoS so that isolates can be sorted by phenotype, allowing rapid identification of linked genomic signatures and the development of prognostic approaches for diagnostic, epidemiology and surveillance. We will develop tools to rapidly collate data for a given strain type and produce a concise phenotypic and clinical profile that provides users with an evidence-based decision-making platform. The Canadian Food Inspection Agency, Health Canada, Agriculture Canada, provincial public health laboratories and the National Microbiology Laboratory-Public Health Agency of Canada group are expected to be end-users of the projects outcomes.

## Future Genomic and Biological Studies of *Salmonella*

We will continuously improve SalFoS by adding *Salmonella* strains, NGS data and analysis as well as experimental results. Another aim of the Syst-OMICS consortium is to avoid duplication of efforts in *Salmonella* genomics and enhance interest from researchers having common goals. Additional members are welcome to join in and expand on our original Genome Canada project. We also intend to seek collaboration with other groups to connect our database with those developed for other *Salmonella* genomes. Finally, the *Salmonella* Syst-OMICS project could be a model for other groups interested in the bacterial genomics of infectious diseases, a strategy that we are also pursuing for *Pseudomonas aeruginosa* (Freschi et al., 2015).

## Author Contributions

J-GER, JJ, LF, IK-I and RL collected strains, performed the analyses and drafted the manuscript. BB provided support for sequencing and analysis. MD contributed to the development of SalFoS. All other authors handled strains and collected metadata. All authors revised the manuscript.

## Conflict of Interest Statement

The handling Editor declared a shared affiliation, though no other collaboration, with the authors ST and AG, and the handling Editor states that the process met the standards of a fair and objective review. The other authors declare that the research was conducted in the absence of any commercial or financial relationships that could be construed as a potential conflict of interest.

## Footnotes

1 https://www.cdc.gov/salmonella/reportspubs/salmonella-atlas/serotypesnapshots.html

2 http://www.pulsenetinternational.org/networks/usa/

3 https://www.nml-lnm.gc.ca/Pulsenet/index-eng.htm

4 http://enterobase.warwick.ac.uk/

5 http://dev.irida.ca/

6 https://github.com/ejfresch/saturnV
