# Supplementary material for: A Syst-OMICS Approach to Ensuring Food Safety and Reducing the Economic Burden of Salmonellosis
Source: Front Microbiol. 2017 Jun 2;8:996. doi: 10.3389/fmicb.2017.00996 (PMC5454079; doi:10.3389/fmicb.2017.00996)
Supplement: Supplementary file 1 [file Presentation_1.PDF]

The *Salmonella* Syst-OMICS project flowchart

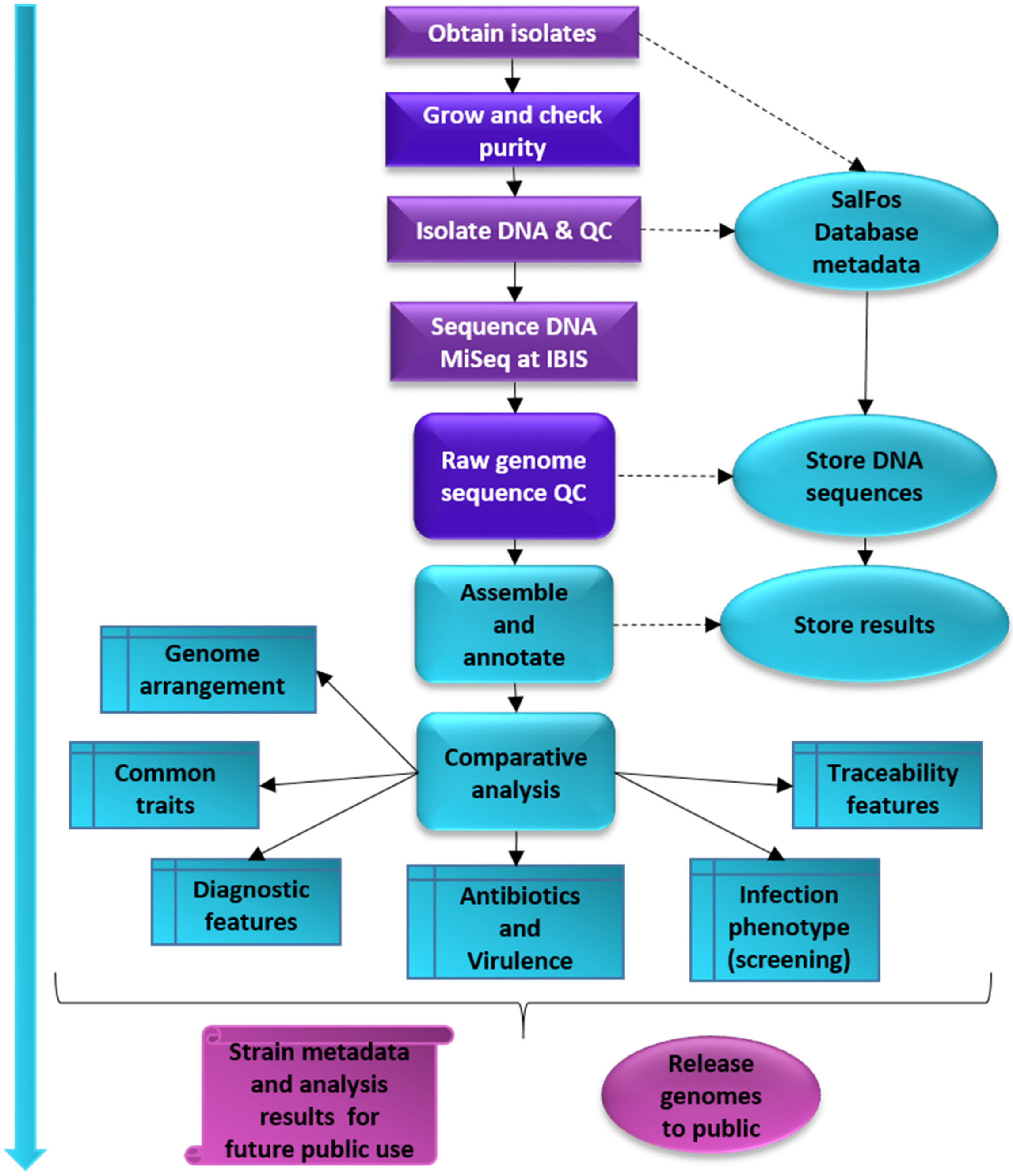

Screening strategy for selection of representative *Salmonella* isolates

The strategy used is a funnel-type multi-host screening system to define levels of virulence starting with 4,500 genome sequenced *Salmonella* isolates. A collection of clinical, non-clinical, environmental and fresh produce isolates will be used for this screening, with a strong focus on fresh produce.

The selection criteria for high-throughput screening (HTS), which can be quantitative or qualitative, include 2 control *Salmonella* isolates, the parent virulent Typhimurium strain SL1344 and its isogenic non-virulent Δ*invA* Δ*sseB* double deletion mutant, and the 10 most frequent *Salmonella* serotypes found in Canada and the US. Additional isolates will be selected based upon the diversity as defined by the phylogeny and metadata found in SalFoS, for a total of 300 isolates. HTS is done using mammalian cell lines by measuring adhesion, invasion and survival of *Salmonella* during interaction with human epithelial cells. *Salmonella* isolates are then classified according to pathogenicity and survival as follows: low ($\log_{10}$ CFU < 4), intermediate ($4 < \log_{10}$ CFU < 5) and high virulence ($\log_{10}$ CFU > 5). HTS is also planned in macrophage and *Acanthamoeba*.

The selection criteria for mice assays can be quantitative or qualitative and will be done using the same control strains and most frequent serotypes as above. In addition, 25 clinical and 75 non-clinical isolates will be screened. Isolates are considered highly virulent when $\log_{10}$ CFUs/gr of tissue is between 7 and 10. Virulence is considered intermediate when $\log_{10}$ CFUs is between 4 and 6 and low when $\log_{10}$ CFUs is below 4.

The gastrointestinal tract models will be done using 5 clinical and 5 environmental isolates.
